# Supplementary material for: Anomalous trends in global ocean carbon concentrations following the 2022 eruptions of Hunga Tonga-Hunga Ha’apai
Source: Commun Earth Environ. 2024 May 10;5(1):247. doi: 10.1038/s43247-024-01421-8 (PMC11087252; doi:10.1038/s43247-024-01421-8)
Supplement: Supplementary file 1 — Reporting Summary [file 43247_2024_1421_MOESM1_ESM.pdf]

nature portfolio

Corresponding author(s): Bryan Franz

Last updated by author(s): Apr 11, 2024

Reporting Summary

Nature Portfolio wishes to improve the reproducibility of the work that we publish. This form provides structure for consistency and transparency in reporting. For further information on Nature Portfolio policies, see our Editorial Policies and the Editorial Policy Checklist.

Statistics

For all statistical analyses, confirm that the following items are present in the figure legend, table legend, main text, or Methods section.

n/a	Confirmed	
☐	☒	The exact sample size (*n*) for each experimental group/condition, given as a discrete number and unit of measurement
☒	☐	A statement on whether measurements were taken from distinct samples or whether the same sample was measured repeatedly
☐	☒	The statistical test(s) used AND whether they are one- or two-sided<br>*Only common tests should be described solely by name; describe more complex techniques in the Methods section.*
☒	☐	A description of all covariates tested
☐	☒	A description of any assumptions or corrections, such as tests of normality and adjustment for multiple comparisons
☐	☒	A full description of the statistical parameters including central tendency (e.g. means) or other basic estimates (e.g. regression coefficient) AND variation (e.g. standard deviation) or associated estimates of uncertainty (e.g. confidence intervals)
☒	☐	For null hypothesis testing, the test statistic (e.g. *F*, *t*, *r*) with confidence intervals, effect sizes, degrees of freedom and *P* value noted<br>*Give P values as exact values whenever suitable.*
☒	☐	For Bayesian analysis, information on the choice of priors and Markov chain Monte Carlo settings
☒	☐	For hierarchical and complex designs, identification of the appropriate level for tests and full reporting of outcomes
☒	☐	Estimates of effect sizes (e.g. Cohen's *d*, Pearson's *r*), indicating how they were calculated

Our web collection on statistics for biologists contains articles on many of the points above.

Software and code

Policy information about availability of computer code

Data collection	For satellite data, no software was required, as all data were collected from open access archives via standard web browser. BGC-Argo float data were obtained using MATLAB and the OneArgo-MAT toolbox Version 1.0 (https://zenodo.org/records/6588042).
Data analysis	Satellite ocean color data processed using l2gen version 9.6 obtained from SeaDAS version 8 (http://seadas.gsfc.nasa.gov/). The vector radiative transfer code used for the simulation is developed and maintained by Dr. Pengwang Zhai of the University of Maryland Baltimore County (UMBC), and is available upon request (pwzhai@umbc.edu). Data analyses and plotting were performed using Interactive Data Language (IDL) version 8.9 (https://www.nv5geospatialsoftware.com/Products/IDL).

For manuscripts utilizing custom algorithms or software that are central to the research but not yet described in published literature, software must be made available to editors and reviewers. We strongly encourage code deposition in a community repository (e.g. GitHub). See the Nature Portfolio guidelines for submitting code & software for further information.

Data

Policy information about availability of data

All manuscripts must include a data availability statement. This statement should provide the following information, where applicable:

- Accession codes, unique identifiers, or web links for publicly available datasets
- A description of any restrictions on data availability
- For clinical datasets or third party data, please ensure that the statement adheres to our policy

All data used in our analysis were acquired from public repositories. The OMPS stratospheric aerosol data is available from NASA's Goddard Earth Sciences Data and Information Services Center (GES DISC, https://disc.gsfc.nasa.gov/).The NASA ocean color data is available from the Ocean Biology Distributed Active Archive Center (OB.DAAC, http://oceancolor.gsfc.nasa.gov). The ESA/EUMETSAT ocean color time-series data from OLCI is available at https://metis.eumetsat.int/data/oc/. The BGC-ARGO in situ bbp data is available from https://usgodae.org/argo/argo.html.

Human research participants

Policy information about studies involving human research participants and Sex and Gender in Research.

Reporting on sex and gender	N/A
Population characteristics	N/A
Recruitment	N/A
Ethics oversight	N/A

Note that full information on the approval of the study protocol must also be provided in the manuscript.

Field-specific reporting

Please select the one below that is the best fit for your research. If you are not sure, read the appropriate sections before making your selection.

☐ Life sciences ☐ Behavioural & social sciences ☒ Ecological, evolutionary & environmental sciences

For a reference copy of the document with all sections, see nature.com/documents/nr-reporting-summary-flat.pdf

Ecological, evolutionary & environmental sciences study design

All studies must disclose on these points even when the disclosure is negative.

Study description	The study compared monthly mean values of satellite remote sensing measurements of ocean biol-optical properties and atmospheric properties for large regions of the ocean, over the year 2022, with the historical monthly mean annual cycles from the same satellite measurements, to demonstrate that anomalies observed in 2022 are a result of the Hunga Tonga volcanic eruptions that invalidate some of the assumptions in the satellite ocean bio-optical property retrieval algorithm.
Research sample	Global and regional monthly mean satellite remote sensing measurements of ocean bio-optical properties and atmospheric aerosols.
Sampling strategy	Monthly mean values sufficient to demonstrate deviation in annual cycle from climatological norm.
Data collection	Data collected from public repositories (NASA, ESA, BGC-Argo).
Timing and spatial scale	Monthly means values for the year 2022 were compared with the historical annual cycles from multiple satellite missions over their mission lifetimes. Data is continuous from start of each mission through 2022, with the earliest mission being MODIS on Terra that launched in 1999. Spatial scales are global, with separation by northern and southern hemispheres to demonstrate that the effect is localized to the southern hemisphere. More detailed analyses focus on a large region around the south pacific gyre, to enable comparison across missions and space agencies and to provide localized viewing and solar geometries to perform an effective simulation of the phenomenon.
Data exclusions	No data excluded within the regions studied.
Reproducibility	All data are freely available and analysis results are readily reproducible.
Randomization	Not applicable.
Blinding	Not applicable.

Did the study involve field work? ☐ Yes ☒ No

Reporting for specific materials, systems and methods

We require information from authors about some types of materials, experimental systems and methods used in many studies. Here, indicate whether each material, system or method listed is relevant to your study. If you are not sure if a list item applies to your research, read the appropriate section before selecting a response.

Materials & experimental systems

n/a	Involved in the study
☒	☐ Antibodies
☒	☐ Eukaryotic cell lines
☒	☐ Palaeontology and archaeology
☒	☐ Animals and other organisms
☒	☐ Clinical data
☒	☐ Dual use research of concern

Methods

n/a	Involved in the study
☒	☐ ChIP-seq
☒	☐ Flow cytometry
☒	☐ MRI-based neuroimaging
